# Prior Roux‐en‐Y Gastric Bypass Patient With Duodenal Fistula After Perforated Duodenal Ulcer Case Report

**DOI:** 10.1155/cris/2055396

**Published:** 2026-02-10

**Authors:** Caroline Couch, Matthew Abourezk

**Affiliations:** ^1^ Allegheny General Hospital, 320 East North Avenue Suite 592, Pittsburgh, Pennsylvania, USA, wpahs.org

## Abstract

Perforated duodenal ulcers are a rare occurrence in patients with a history of Roux‐en‐Y gastric bypass (RNYGB). This case report is of a patient who had two separate presentations of perforated duodenal ulcer despite a history of RNYGB and who progressed to a high‐output duodenal‐cutaneous fistula after failing the second Graham patch. This highlights the impact ulcer disease can have even in RNYGB patients.

## 1. Introduction

With the steady increase in obesity in the United States and globally, there are more and more patients who have turned to bariatric surgery, including the Roux‐en‐Y gastric bypass (RNYGB). The bypass creates a small gastric pouch and diverts ingested food into the jejunum, bypassing the stomach and proximal small bowel in a method that has both restrictive and metabolic impacts on weight loss. While rare, peptic ulcer disease (PUD) in the excluded stomach and duodenum does still occur, and several case reports and small case series have been written [1, 2]. A review from 2020 of these patients cites a cure rate of 100% of perforated duodenal ulcers with either Graham patch or remnant gastrectomy [3]. This case shows a patient who not only had a recurrent duodenal ulcer perforation but also failed Graham patch and then declined further intervention, highlighting the natural course of the disease pathology.

## 2. Case Presentation

A 58‐year‐old female patient with a history of RNYGB and recent total knee replacement as well as anemia, anxiety, and post‐traumatic stress disorder initially presented to the emergency department for epigastric pain and nausea. She denied aspirin, nonsteroidal anti‐inflammatories (NSAIDs), and smoking at home but did get NSAIDs and a dose of steroids during her recent admission for knee replacement. Computed tomography (CT) scan showed inflammation of the first portion of the duodenum as well as adjacent free fluid. She was taken to the operating room, where duodenal perforation was confirmed, and a modified Graham patch was performed, which passed a leak test on admission but was later complicated by a post‐op intra‐abdominal abscess treated with interventional radiology drainage.

Two years later, the patient was admitted for a humerus fracture after a fall. This underwent fixation, but she then developed abdominal pain, hypotension, and fever. CT showed free fluid and free air. Due to her medical instability, she was taken for a laparotomy with modified Graham patch repair just distal to her prior repair. This admission was complicated by bacteremia, fascial dehiscence, post‐op leak, and malnutrition requiring total parenteral nutrition (TPN). She was considered a poor surgical candidate for further operations and was managed medically. Bilious drainage from the midline wound stopped, and she was eventually able to stop TPN and resume a bariatric diet in the following months. However, she returned with evidence of a high‐output fistula. TPN was again started, and output decreased until she stopped the TPN and began eating again, which again increased her output.

Due to social stressors and difficulty with her mental health, she declined further treatment with TPN. The patient’s last follow‐up was 7 months after her second Graham patch surgery, at which time she had persistent fistula output. No imaging ever showed a gastrogastric fistula despite the ongoing enterocutaneous fistula. The fistula never drained anything other than bile, even with the oral methylene blue challenge. *Helicobacter pylori* was negative during both perforations.

## 3. Discussion

Duodenal PUD is a rare occurrence in patients with a history of RNYGB and even more rarely results in a duodenal ulcer perforation. However, there are cases that have been documented [1–3]. In a review of PUD after RNYGB, 100% of the duodenal perforations were cured with oversewing, duodenostomy, Graham patch, or gastric remnant resection [3]. There is no mention of a duodenal fistula after any of these reported cases except when one was intentionally created for surgical drainage [1, 2] and which healed despite the patient taking oral nutrition. While this is a single case report, it does show a previously undocumented occurrence with a duodenal perforation that did not heal with initial surgical treatment and continues to have duodenal fistula output while taking oral nutrition. Free air is an uncommon finding from perforation when the limb is fully excluded [3]. Given that she did have free air on both presentations and had such a high‐output fistula with oral intake, this would suggest an undiagnosed gastrogastric fistula existed, but none of her CT scans were able to confirm this, and no ingested methylene blue was seen leaking from her abdominal wound. Because she had a recurrent ulcer and a high‐output fistula, this patient likely would have benefited from a remnant gastrectomy for acid reduction and removal of any possible gastrogastric fistula as recommended by Macgregor et al. [1] at which time the area of the fistula source could have also been resected, but the patient was initially unstable and later had poor mental health and compliance with medical treatment, which precluded a second surgery. As for the etiology of her perforation, she did not have common risk factors, including *H. pylori* or smoking. However, in the case of both her perforations, she was in the immediate postoperative period after an orthopedic procedure. During both of those hospitalizations, she was given NSAIDs and was given a dose of steroids prior to the first perforation. NSAIDs are a known risk factor for PUD, though they were associated with only 15% of the PUD in RNYGB patients in an existing review [3]. Of note, she was never tested for Zollinger‐Ellison syndrome, which should be included in the differential diagnosis given her recurrent ulcers.

## 4. Conclusion

This case report illustrates that not only can duodenal ulcers perforate in patients with a history of RNYGB, but they can also result in a high‐output fistula in a diverted portion of the bowel. Further research into the existence and management of duodenal fistulas from perforated PUD in RNYGB patients is warranted given this is a single case report and the patient declined further treatment of her fistula.

## Funding

This research did not receive specific funding.

## Conflicts of Interest

The authors declare no conflicts of interest.

## Data Availability

Data sharing is not applicable to this article, as no new data were created or analyzed in this study.

## References

[1] Macgregor A. M. C. , Pickens N. E. , and Thoburn E. K. , Perforated Peptic Ulcer Following Gastric Bypass for Obesity, The American Surgeon. (1999) 65, no. 3, 222–225, 10.1177/000313489906500307. 10075296

[2] Iskandar M. E. , Chory F. M. , Goodman E. R. , and Surick B. G. , Diagnosis and Management of Perforated Duodenal Ulcers following Roux-en-Y Gastric Bypass: A Report of Two Cases and a Review of the Literature, Case Reports in Surgery. (2015) 2015, 1–4, 10.1155/2015/353468, 353468. PMC4408622 25949843

[3] Plitzko G. , Schmutz G. , Kröll D. , Nett P. C. , and Borbély Y. , Ulcer Disease in the Excluded Segments After Roux-en-Y Gastric Bypass: A Current Review of the Literature, Obesity Surgery. (2021) 31, no. 3, 1280–1289, 10.1007/s11695-020-05123-w. 33230760 PMC7921036

